# Adaptation and ecological speciation in seasonally varying environments at high latitudes: *Drosophila virilis* group

**DOI:** 10.1080/19336934.2021.2016327

**Published:** 2022-01-21

**Authors:** Anneli Hoikkala, Noora Poikela

**Affiliations:** Department of Biological and Environmental Sciences, University of Jyväskylä, Jyväskylä, Finland

**Keywords:** Local adaptation, circadian clock, photoperiodic timer, reproductive diapause, cold tolerance, reproductive barriers, genome sequencing, candidate genes, chromosomal inversions

## Abstract

Living in high latitudes and altitudes sets specific requirements on species’ ability to forecast seasonal changes and to respond to them in an appropriate way. Adaptation into diverse environmental conditions can also lead to ecological speciation through habitat isolation or by inducing changes in traits that influence assortative mating. In this review, we explain how the unique time-measuring systems of *Drosophila virilis* group species have enabled the species to occupy high latitudes and how the traits involved in species reproduction and survival exhibit strong linkage with latitudinally varying photoperiodic and climatic conditions. We also describe variation in reproductive barriers between the populations of two species with overlapping distributions and show how local adaptation and the reinforcement of prezygotic barriers have created partial reproductive isolation between conspecific populations. Finally, we consider the role of species-specific chromosomal inversions and the X chromosome in the development of reproductive barriers between diverging lineages.

## Introduction

At high latitudes and altitudes, climatic conditions show remarkable seasonal and spatial variation, as well as major environmental perturbations that can change species distribution, create isolated refugia and lead to secondary contacts between newly evolved taxa [1]. Adaptation into this kind of environment sets specific requirements on insects’ ability to forecast seasonal changes on the basis of photoperiodic cues and to prepare for the winter by entering diapause, decreasing locomotor and feeding activity and going through various metabolic changes that increase cold tolerance. Accordingly, different photoperiodic and climatic conditions experienced by individuals at their home site have created latitudinal clines in the timing and intensity of egg, larval or adult diapause and cold tolerance in several insect species with a wide geographic range [2–5].

Ecological divergence is suggested to be the earliest stage of speciation in lineages that are in contact with each other, since no taxa can live in exactly the same ecological niche [6]. Ecological speciation may be induced by directional natural and sexual selection on one or more traits, if climatic factors or interactions with closely related species induce variation in sexually selected traits and associated preferences and generate partial premating isolation between conspecific populations [7]. These kinds of ‘magic traits’ can facilitate speciation especially in the presence of gene flow [8]. Good examples of magic traits are the wing melanization, which functions both in thermoregulation and species recognition [9], and the chemosensory system, which can play a crucial role in resource- and stress-mediated adaptation, mate choice and speciation [10]. Moreover, chromosomal inversions can enhance population divergence in the presence of gene flow by reducing recombination between locally adapted allele complexes within inverted chromosomal regions [11,12], and they may also maintain polymorphisms in these complexes within the species [13]. For example, in *D. melanogaster* seasonally fluctuating polymorphisms are enriched in large chromosomal inversions, and the direction of allele frequency changes at these polymorphisms can be predicted by weather conditions in the weeks prior to sampling, which nicely links the environment and the genomic response to selection [13].

In this review, we go through recent and ongoing studies on the northern and high-altitude *Drosophila virilis* group species whose distribution ranges over several latitudes and/or which have populations at high altitudes. We pay special attention to an important role of insect time-measuring systems in adaptation to seasonally varying photoperiodic and temperature conditions prevailing at high latitudes and to strong linkages that the traits involved in species survival and reproduction show with the climatic conditions at different latitudes. Next, we go through studies on the reproductive barriers between northern *virilis* group species, and on the role of male courtship songs in sexual selection and species recognition. We also describe variation in reproductive barriers between the populations of two species with overlapping distributions (partial sympatry) and show how adaptation into local climatic conditions and/or the reinforcement of reproductive isolation sympatric populations have increased divergence in species’ courtship cues and induced reproductive barriers between conspecific populations living within and outside the area of sympatry. Finally, we show that the chromosomal inversions and the X chromosome have played a prominent role in adaptation and speciation processes in two partly sympatric *virilis* group species. Overall, the review emphasizes the value of northern and high-altitude insect species in helping to understand the specific features that adaptation into harsh, seasonally varying environments has required.

## Evolutionary history of the *virilis* group

The *virilis* group species offer great material for adaptation and speciation research, because many of them are distributed in areas with highly variable photoperiodic and climatic conditions and occur sympatrically in some parts of their distribution, and because the species possess multiple sexually selected signals and chromosomal inversions. The *virilis* group was most likely established in the temperate forest of East Asia [14], while the last common ancestor of the group probably had a Holarctic distribution from which the North American and the Eurasian lineages evolved as a result of a vicariant event [15]. Morales-Hojas et al. [15] divided the group into *virilis* phylad and *littoralis, kanekoi* and *montana* subphylads on the basis of a few nuclear and mitochondrial ribosomal RNA genes. They estimated the split between the *montana* subphylad and other lineages to have occurred close to 10 Mya (million years ago), and the split between the *virilis* phylad and the *littoralis* and *kanekoi* subphylads ~8.5 Mya. More recently, Yusuf et al. (in prep.) identified 1336 single-copy orthologs from genome data to produce a phylogeny with strong support. This phylogeny resolved three groups: the *virilis* phylad (*D. virilis, D. americana, D. novamexicana* and *D. lummei* [*D. texana* was not sampled]), the *littoralis* phylad (*D. littoralis, D. ezoana* and *D. kanekoi*) and an earlier branch leading to the *montana* phylad (*D. montana, D. flavomontana, D. borealis* and *D. lacicola*). Yusuf et al. (in prep.) suggested that based on putatively neutral intron sequences, the *montana* phylad has diverged from other lineages around 9 Mya and the *virilis* and *littoralis* phylads from each other around 7.5 Mya. If correct, this is consistent with a Pliocene origin of the phylads. Yusuf et al. [in prep.] also found evidence of both ancient and recent gene flow within the phylads.

**Figure 1.**
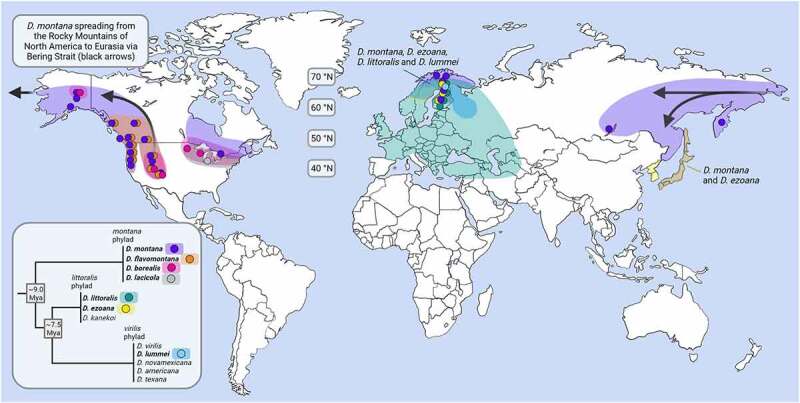
Geographic distributions of the *virilis* group species spread on high latitudes and/or on high altitudes, marked in bold in the simplified phylogeny of the group (the basal nodes for the diverge of the *montana, littoralis* and *virilis* phylads are based on Yusuf et al., in prep.). *D. lummei, D. littoralis* and *D. ezoana* are suggested to be born in Eurasia and *D. montana, D. flavomontana, D. borealis* and *D. lacicola* in North America. *D. montana* has later on spread from North America to Eurasia through the Bering Strait (see the arrows). Species distributions are based on the maps presented in Throckmorton [14], and more recent collections made by us or our colleagues (our most important collecting sites are marked with dots). The figure was created with BioRender.com.

Throckmorton [14] gives detailed information on the distribution and habitat requirements of the *virilis* group species, and we concentrate here on the species distributed at high latitudes and/or altitudes (see Figure 1). *D. lummei* from the *virilis* phylad and *D. littoralis* and *D. ezoana* from the *littoralis* phylad have spread to high latitudes in Asia and Europe (*D. lummei* has become extremely rare throughout its distribution and the present distributions of *D. littoralis* and *D. ezoana* in Asia are not known). Among the four species of the *montana* phylad, three – *D. lacicola, D. borealis* and *D. flavomontana* – are found only in North America, *D. borealis* and *D. flavomontana* having populations at a wide range of altitudes (<2800 m) and latitudes (see Figure 1). The fourth species of this phylad, *D. montana*, is distributed around the northern hemisphere, but its present existence in the central parts of North America and in Siberia is unknown. In North America, *D. montana* is partly sympatric with *D. flavomontana* and *D. borealis*, and possibly with *D. lacicola*, and in northern Europe with *D. littoralis, D. ezoana* and *D. lummei*. Estimates of the divergence time of the North American and European *D. montana* populations vary from 450,000–950,000 years [16,17] to 1 750,000 years [18]. Thus, it is plausible that *D. montana* spread from the Rocky Mountains of North America to Alaska soon after reaching species status, possibly spending some time in Berengia (far north-eastern Siberia and north-western North America) and spreading from there through Asia to northern Europe (see arrows in Figure 1). Land connection between North America and Asia has been open at least in the time periods between 920,000 and 870,000, between 140,000 and 130,000 and between 50,000 and 15,000 years ago [20,21], and apparently large pieces of land have remained unglaciated during these periods [20].

## Circadian clock system and the photoperiodic timer of the *virilis* group species show unique features that are adaptive at high latitudes

**Figure 2.**
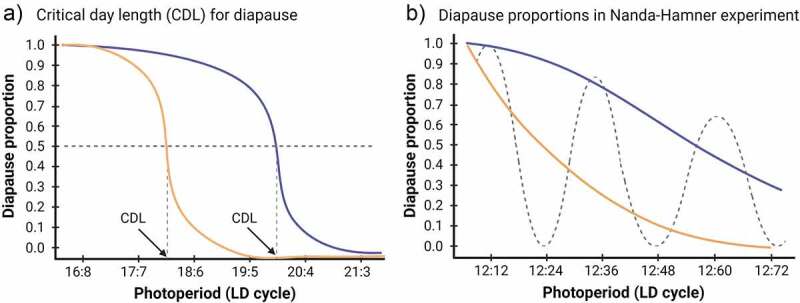
Schematic curves illustrating changes in female diapause proportion in *D. montana* strains from Southern (Orange) and Northern (blue) Finland in experiments measuring the critical day length (CDL) for diapause induction (a) and in Nanda–Hamner (NH) experiments examining the presence of circadian components in the photoperiodic time-measuring system (PPTM) and the stability of diapause induction against perturbations in photoperiod length (b). (a) CDLs correspond the photoperiods where 50% of the females of each strain enters diapause (17,5:6,5 for the Southern strain and 19,5:4,5 for the Northern strain). (b) Diapause proportions for the same strains in NH experiment in the photoperiods consisting of 12 h light and 12–72 h dark. A dashed line with high diapause peaks in circadian photoperiods (12:12, 12:36 and 12:60) illustrates cyclic changes in diapause proportions in the presence of slightly damping circadian oscillation (positive NH-response). Blue and orange lines illustrate the negative NH-responses (no cyclicity in female diapause proportion) of the northern and southern *D. montana* strains, respectively (northern strains show higher diapause proportions under extralong photoperiods). The figure was created with BioRender.com.

Insects living in high latitudes need to adapt to exceptional daily and seasonal variation in photoperiodic and temperature conditions. The winters are dark (especially under the snow cover), the summers are characterized by the midnight sun, and day lengths can vary by up to 1 h per week during autumn and spring. Continuous darkness and light set specific requirements for insect daily activity rhythms in winter and mid-summer. On the other hand, clear and continuous changes in day length (photoperiod) during the other times of year offer a reliable cue for the forthcoming seasonal changes, helping insects to adjust their life cycle according to the annual rhythm. Accordingly, northern insect species, which spend the winter in diapause, typically enter this stage when the day length decreases below a critical day length in late summer/early autumn [22,23]. The critical day length (CDL), also called the critical night length (CNL) or the critical photoperiod (CPP), is defined as a photoperiod where half of the eggs, larvae or adult females of a given population enters diapause (see Figure 2(a)).

Insect ability to trace daily and seasonal changes in photoperiod is based on two time-measuring systems, the circadian clock system measuring day length and the photoperiodic time-measuring system (PPTM) underlying seasonal changes in traits like reproduction and migration. Nearly all organisms have evolved endogenous self-sustained timekeeping mechanisms (maintained without external stimuli) to track and anticipate cyclic changes in the environment, but the strength of connection between the circadian clock system and PPTM seems to vary between the species. In the northern drosophilids, the circadian clock is either not self-sustained or is uncoupled from PPTM, which might be adaptive for animals colonizing weakly rhythmic environments [24].

### Circadian clock system

Circadian clock system regulates daily rhythms in insect mating, oviposition, eclosion and locomotor activity, but is also involved in various kinds of cellular processes from neurotransmitter secretion to metabolism [25]. Circadian rhythms can persist for a long time even in constant conditions, but as these ‘free-running rhythms’ often deviate from ~24 h in constant light or darkness, a circadian pacemaker has to be resynchronized every day by environmental signals [26].

In *Drosophila* species, the central circadian clock pacemaker resides in neurons associated with the visual centre of the brain, and the core clock genes regulate membrane excitability in master pacemaker neurons generating molecular circadian rhythms. In *D. melanogaster*, this pacemaker involves approximately 150 circadian clock cells, which express the core clock genes and form an extensive neuropeptidergic network in seven neuronal clusters in the brain controlling rhythmic behaviours that define the fly’s daily activity profile [27,28]. The photopigment cryptochrome (CRY) and the neuropeptide pigment dispersing factor (PDF) are generally co-expressed in two clusters of small and large ventrolateral clock neurons (s-LNvs and l-LNvs), respectively. PDF-positive s-LNvs are essential for flies’ morning activity (M cells) under cycling environmental conditions and for their rhythmic activity under constant darkness, while the PDF-negative s-LNv and dorsolateral neurons (LNds) are responsible for oscillators controlling evening activity (E cells) [29]. However, proper interaction between all clock cells is important for adapting the flies’ activity to different photoperiods [30]. At the molecular level, the function of the *D. melanogaster* clock is based on two feedback loops between the core clock genes, one involving the clock genes *period* (*per), timeless* (*tim), cycle* (*cyc*) and *Clock* (*Clk*), and the second one involving *cycle* (*cyc), Clock* (*Clk), vrille* (*vri*) and *PAR Domain Protein 1ε* (*Pdp1ε*) [28].

In contrast to *D. melanogaster*, the flies of the high-latitude *virilis* group species show only evening activity and retain their free-running eclosion and locomotor activity rhythms better in constant light than in darkness [30,31–33]. In these species, CRY is not expressed in l-LNvs and PDF is expressed in some cells located in the dorsal brain instead of s-LNvs neurons, which can at least partly explain the lack of flies’ morning activity and their higher rhythmicity in constant light than in darkness [34,31,32,35]. According to Beauchamp et al. [36], this kind of CRY/PDF expression has only been found in the species of the *virilis-repleta* radiation that colonized high latitudes. Moreover, circadian clock genes of the *virilis* group species show high divergence from those of other *Drosophila* species [37], and in *D. montana* the peak expression levels of *per* and *tim* are not locked to lights-off transition in any photoperiod like in *D. melanogaster* [30]. Overall, rhythmicity of the northern *virilis* group flies in constant light is adaptive to the long (even continuous) days during the fly mating season in early summer, while their arrhythmicity in darkness is adaptive during the long and dark winters when the flies are in an inactive state [31].

### Photoperiodic time-measuring system (PPTM) underlying diapause and its connection with the circadian clock

PPTM enables insects to anticipate the forthcoming seasonal changes in their environment and to prepare for them in advance. PPTM underlying reproductive diapause involves two processes: the detection of qualitative differences between long 300 and short days/nights (photoperiodic clock) and the accumulation of quantitative information on photoperiods (photoperiodic counter) up to an internal threshold at which the induction of diapause is complete [38]. In an hourglass model, the night length measurement is suggested to be based on the accumulation of a hypothetical chemical substance (‘diapause titer’) during the dark period [39], while in other models, PPTM is expected to rely on circadian rhythms driven by non-damping or damping circadian oscillator(s) operating either at night length measurement or at the counter level. In the internal coincidence model, PPTM is based on seasonal changes in the phase relationship between the morning (M) and evening (E) oscillators, and thus it can function only in species like *D. melanogaster* that show both morning and evening activity. Finally, in the basic external coincidence model, the night length is defined as long or short depending on whether the photoinducible phase, oscillating in ~24 h cycle, coincides regularly with a light or a dark period [38]. However, in the quantitative versions of this model, the evaluation of long night cycles is suggested to rest on the accumulation of a diapause titre like in the hourglass model [40]. At high latitudes, a highly damped hourglass-like timer may synchronize more easily to rapidly lengthening autumnal nights than circadian mechanism [41].

We have used Nanda–Hamner (NH) protocol to find out whether the PPTM regulating reproductive diapause in *D. montana* involves circadian components. In this protocol, the proportion of females giving a short-day response (e.g. entering diapause) is typically measured in a set of experiments with the day length of 12 h and the night length varying from 4 to 72 h (see Teets and Meuti [42]). *D. montana* females show no circadian rhythmicity (high diapause peaks in photoperiods with a length of 24 h or its multiples; Figure 2(b)) in their diapause proportions in NH experiments, which suggests that the PPTM of this species is either based on heavily damping circadian oscillator(s) or that it lacks strong oscillators [43,44]. NH experiments have also shown that *D. montana* females measure the night length quantitatively, that their photoperiodic counter may play a slightly different role in extra short and long photoperiods and that the northern strains of this species show high stability against perturbations in photoperiod length and in the presence of light: dark (LD) cycles [45], see Figure 2(b). Accordingly, the function of PPTM in *D. montana* is best explained by the quantitative versions of the damped external coincidence model [45].

Lankinen [46] studied the connection between the critical day length (CDL) for diapause induction (regulated by PPTM) and the properties of the free-running eclosion rhythm (regulated by circadian clock) in *D. littoralis* strains from the latitudes between 40°N and 70°N, and found statistical correlations between these traits. However, when performing crosses between the northern and southern *D. littoralis* strains over 54 generations, Lankinen and Forsman [47] found that the flies’ photoperiodism (diapause) and circadian rhythm (eclosion) are regulated by different, but closely linked genes. Moreover, Kauranen et al. [43] performed quasi-natural selection on *D. montana* diapause behaviour by transferring the progeny flies in each generation into a shorter day length. Nine generations of selection changed CDL, the frequency of diapausing females under non-circadian photoperiods in NH experiments and fly cold tolerance towards the phenotypes typical of lower latitudes, but had no effect on the period of fly locomotor activity rhythm. This gives support to the view that the evolution of longer CDLs at high latitudes is driven by selection from longer summer days directly on CDL [48] rather than through the circadian clock system [49].

## Adaptation of the northern *virilis* group flies into seasonally changing environmental conditions

### Fly life-cycle and mate choice in the wild

**Figure 3.**
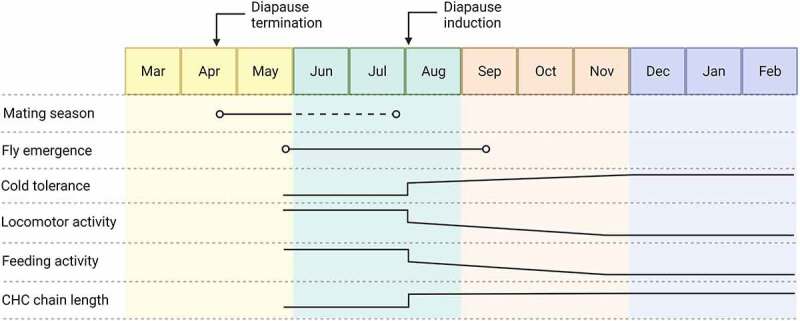
Mating season and offspring emergence of overwintered *D. montana* flies in the wild and changes in behavioural and physiological traits occurring when the females enter diapause (differences between the females emerged before and after CDL) and towards the winter in environmental conditions typical to northern Finland. Diapause induces changes in female cold tolerance, locomotor and feeding activity and the length of cuticular hydrocarbon chains (CHCs) (see the arrow marking the CDL for diapause induction). Moreover, females’ cold tolerance increases and their locomotor and feeding activity decrease gradually towards the winter (same kind of changes can be seen also in males). The figure was created with BioRender.com.

Aspi et al. [50] studied the life-cycle of northern *virilis* group species (*D. montana, D. littoralis, D. ezoana* and *D. lummei*) in Kemi, northern Finland, and found the flies to have a short but intense mating period in spring/early summer after the termination of diapause. During this period, and to a lesser extent in mid and late summer, the flies gathered on food patches on sap flows, rotting plant material and/or partly barren tree trunks, where the courtship and mating took place. Overwintered flies came to food baits from late April/early May to the beginning of July and the first flies of the summer generation in late June and early July. After the first summer emergence peak, there was a slight decline in fly numbers followed by an increase in late summer/early autumn. In late summer, the malt baits attracted mainly sexually mature females, while the diapausing flies of both sexes were resting below a wooden bridge, paying no attention to each other [50]. Figure 3 depicts a calendar showing the mating season and offspring emergence of overwintered *D. montana* flies in the wild in Northern Finland, and changes occurring in the females of summer generation in important behavioural and physiological traits during diapause and towards the winter.

Spring mating season allows females to choose males, which have survived the winter in good condition, as their mating partner. When comparing the songs of mating *D. montana* and *D. littoralis* males with those of the random males collected at the same time and place before and after overwintering, Aspi and Hoikkala [51] found the females of both species to prefer males producing a courtship song with short sound pulses. In both species the shifts in song characters during overwintering were opposite to those caused by sexual selection, implying a possible balance between sexual and natural selection. Interestingly, *D. montana* females get indirect benefits from mating with a male with short sound pulses/high song frequency, as the quality of male song correlates with the survival rate of his progeny from egg to adulthood, but not with the fecundity of his mating partner [52]. In this species, the quality of sexually selected song traits deteriorates with male age in concert with the decrease in male reproductive success also in laboratory [53].

### Adult reproductive diapause plays a central role in fly overwinter survival

Reproductive diapause is an essential part of the life-cycle of several northern insect species [23]. Diapausing females have undeveloped ovaries and they are not able to produce progeny during the ongoing season, but they gain resource allocation trade-offs in terms of higher stress resistance, slower ageing and higher overwinter survival [54,55]. The timing of diapause is under strong local selection pressures determined, e.g. by the length of the growing season, generation time and insect stress tolerances. Females that enter diapause too early may use part of their energy reserves already before the cold period, while the females that develop ovaries too late may not survive over the cold period and/or may produce progeny that fail to collect enough energy reserves before the winter [56].

In the northern *virilis* group species, female reproductive diapause relies mainly on photoperiodic cues, lasts for up to 9 months and is much deeper and less sensitive to temperature than in *Drosophila* species living at lower latitudes [23]. For comparison, in *D. melanogaster*, diapause continues for 6–7 weeks under 10:14 light:dark (LD) cycle at 12°C and is terminated rapidly after transfer to higher temperature or to long days [57]. *D. montana* males enter diapause under the same CDL as the females, but their inability to inseminate females is highly reversible [58]. The diapausing *D. montana* flies of both sexes have more long-chain and less short-chain hydrocarbons on their cuticle (CHCs) than the reproducing ones (Figure 3), which presumably increases their survival under stressful conditions, but at the same time decreases their attractiveness [58].

The sensitive period, during which external cues can trigger the switch from reproductive stage to diapause, or vice versa, can end at a particular age or last indefinitely, depending on the species. In *D. montana* females, this period starts after emergence [59], and diapausing *D. montana* females develop ovaries within a few days when transferred from short- to long-day conditions [60]. Recently, Lankinen et al. [in prep.] also found that the reproducing *D. montana* females can resorb their ovaries and enter diapause in 1–3 weeks, when transferred from long to short-day conditions, which may explain the lack of sexually mature females in the wild in late summer detected by Aspi et al. [50]. In genus *Drosophila*, a switch from full reproductive stage to diapause as a response to short-day length has earlier been reported only in *Drosophila testacea* [61].

The switch to reproductive diapause is accompanied by various kinds of changes in gene expression. In *D. montana, D. littoralis* and *D. ezoana*, changes in some genes are phase-specific, but a few genes, including *Hsc70, Jon25Bi* and *period*, remain upregulated throughout the diapause [62]. Kankare et al. [63] have also shown diapausing and non-diapausing *D. montana* females reared under the CDL to have large differences in the expression patterns of genes involved with metabolism, fatty acid biosynthesis and metal and nucleotide binding. In this study, differently expressed genes included *myosin, actin* and *cytochromeP450*, which have been previously associated with diapause, as well as genes involved in cuticular hydrocarbon (CHC) synthesis or regulation (*desat1* and *desat2*), acyl-CoA Δ11-desaturase activity (*CG9747*) and odorant binding (e.g. *Obp44A*). Moreover, Parker et al. [64] found the expression level of many genes associated with *D. montana* flies’ reproduction to change in different day lengths in both reproducing and diapausing females, which suggests that the females use day length to cue changes in reproduction both before and after entering into diapause. This fits well with the indefinite sensitive period of *D. montana* females for diapause induction detected by Lankinen et al. [in prep.]. Finally, the quasi-natural selection experiment, where emerging *D. montana* flies were transferred in each generation into a shorter day length, induced extensive divergence between the selection and control line replicates in SNPs associated with 16 gene clusters [43]. The list of genes in these clusters included, e.g. G protein-coupled receptors that function in neuropeptide signalling and neurotransmitter synthesis or reception, as well as genes in insulin signalling and 20-hydroxyecdysone and juvenile hormone pathways that play a central role in diapause induction.

### The virilis group flies show high tolerance to low temperatures

Cold tolerance includes several traits, which can be defined by measuring insect chill coma temperature (CT_min_), lethal temperature (LTe50), lethal time at low temperature (LTi50), chill coma recovery time (CCRT) and/or supercooling point (SCP) [65]. When measuring the cold tolerance of 14 *Drosophila* species, Andersen et al. [65] found the cold tolerance of *D. montana* to be extremely high. For example, while the LTi50 at −2°C ranged in other species from 2 to 408 h, *D. montana* flies were still all alive after 624 h in this temperature. Also, *D. montana* and *D. obscura* were the only species having CT_min_ below 0°C, and *D. montana* flies recovered from chill coma about 50% faster than the next cold-tolerant species, Andersen et al. [65] reported that SCP of *D. montana* is ca. −22°C, which means that the flies can stand this temperature without freezing.

Insects can also use environmental signals such as shortening day length and gradually decreasing temperature to trigger seasonal cold acclimation, and they may cope with sudden cold snaps and regularly occurring diurnal temperature drops through rapid cold-hardening [66,67]. Moreover, Parker et al. [68] found the investment in immune function of *D. montana* flies to be reduced in colder temperatures, which suggests that bacterial pathogens of these flies may be less prevalent or less virulent during cold periods (see Fergusen et al. [69]). In *D. montana*, diapausing females have a higher cold tolerance than the non-diapausing ones, and the females also show seasonal cold acclimation when maintained in low temperature or short-day conditions [43,70,71]. When tracing seasonal changes in the cold tolerance and metabolomic profiles of *D. montana* flies in daily and seasonally changing thermo- and photoperiods, Vesala et al. [71] found fly cold tolerance to increase notably in late autumn and to remain high until late spring, as shown in Figure 3. The levels of fly glucose, trehalose and proline storages were at a moderate level already in autumn and stayed at high levels until the spring, while the myo-inositol concentration increased more than 400-fold during the winter. According to Toxopeus et al. [72], these metabolites may contribute to freeze tolerance via a combination of unique and overlapping non-colligative mechanisms. When investigating transcriptional differences between *D. montana* and *D. virilis* during cold acclimation, Parker et al. [73] found the majority of genes that are differentially expressed during cold acclimation to differ between the species, even though the biological processes associated with them included metabolism, cell membrane composition, circadian rhythms and rhodopsin pathway in both species. Interestingly, one of the genes that was upregulated in response to cold acclimation of 6 days at +5°C in both species was *myo-inositol-1-phosphate synthase* (*Inos*), which encodes the enzyme myo-inositol-1-phosphate synthase. Vigoder et al. [74] tested the role of *Inos* in the cold tolerance of *D. montana* flies using an RNA interference (RNAi) approach, and found its inactivation to increase flies’ temperature-sensitive mortality rate of over 60%, but to have no effect on their CCRT. Poikela et al. [4] also traced the role of a circadian clock gene *vrille* in regulating females’ cold tolerance and cold acclimation ability by silencing it with RNAi in *D. montana*. This study showed that *vrille* plays an important role in CT_min_ and in female cold acclimation ability, which highlights the importance of this gene, and possibly the whole circadian clock system, in enhancing their cold tolerance both during long-term cold acclimation and the rapid cold hardening.

### Species of the *virilis* group are sensitive to high temperatures

According to Parrat et al. [75], distributions of *Drosophila* species may be restricted by the effects of high temperatures on male fertility rather than on fly survival. The authors found the global distributions of 43 species to match better with male-sterilizing temperatures than the upper lethal temperatures. In the cold-tolerant *virilis* group species, male fertility is extremely sensitive to high temperatures, and, e.g. *D. flavomontana* males become sterile in ~32°C and die in ~36°C [75]. Parratt et al. [75] emphasize that temperature-driven fertility losses may be a major threat to biodiversity during climate change and that due to the effects of high temperature on male fertility the available habitat for *D. flavomontana* will have reduced by up to 62.9% by 2080.

## Reproductive diapause, cold tolerance, body colour and size and voltinism show latitudinal variation adjusted by local environmental conditions

**Figure 4.**
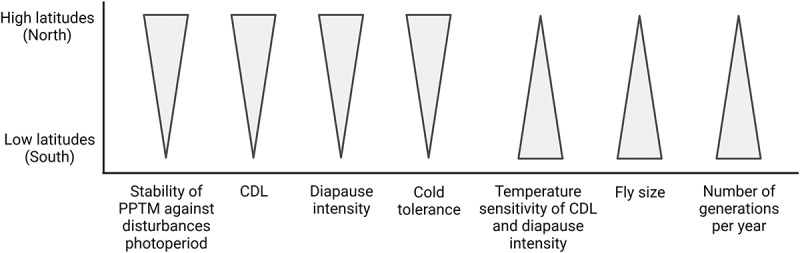
Direction of latitudinal variation in traits important in fly survival and reproduction in *D. montana*. The stability of the photoperiodic time-measuring system (PPTM) against disturbances in photoperiod, the timing (CDL) and intensity of reproductive diapause and cold tolerance increase towards North, while the temperature sensitivity of CDL and diapause intensity, fly size and the number of generations per year (voltinism) increase towards South. The figure was created with BioRender.com.

Insect populations living in different latitudes and altitudes meet different abiotic and biotic conditions around the year, and they may be expected to show variation in several traits affecting fly life cycle and overwinter survival. Thus, it is important to gather several independent sources of evidence, including sibling species, multiple populations or geographic regions and environmental correlations that account for population structure, when studying case for clinal adaptation [76]. A summary of the direction of latitudinal variation in traits important in fly survival and reproduction in *D. montana* is shown in Figure 4.

### Latitudinal variation in the timing of reproductive diapause

Correct timing of reproductive diapause plays a crucial role in fly survival and progeny production, and CDL shows steep latitudinal clines in several insect species [46,77]. This trait is affected by two opposite selection forces, one favouring the females with a short CDL (reproduction in late summer) and the other one favouring the females with a long CDL (early diapause entry). Hairston et al. and Taylor and Spalding [78,79] have suggested that when variation in the onset of cold winter season is relatively low, the superior strategy for insects is to enter diapause at a constant period before this date. In high-latitude populations with great thermal variability, natural selection favours broad ‘safety margins’ so that diapause is initiated when the days are still relatively long, while in the low-latitude populations, the strong time stress for producing several generations per year may lead to reduced sensitivity to photoperiod and postpone the diapause towards the autumn [80].

CDL of *D. montana* shows robust latitudinal variation in northern Europe in spite of relatively high gene flow between populations [81]. This trait also remains constant in fly strains regardless of the number of years that the strains have been maintained in continuous light in laboratory [82]. *D. montana* possesses latitudinal clines on different continents in CDL and female susceptibility to enter diapause (diapause incidence), as well as in the temperature sensitivity of these traits [5]. Northern strains possess longer CDLs than the southern ones, which means that they enter diapause under longer days and at an earlier calendar time than the southern strains (see Figure 2(a)). CDL of *D. montana* decreases by about one hour per five degrees decline in latitude, and a few degrees temperature increase decreases CDL and postpones the diapause to later calendar time, especially in southern populations [5]. Moreover, CDL of this species is affected by climatic factors determining the growing season length at local scale [5].

*D. montana* also shows latitudinal variation in the properties of the photoperiodic time-measuring system (PPTM) underlying reproductive diapause. In Nanda–Hamner (NH) experiment, the females of the northernmost strains enter diapause under both circadian (24 h and its multiples) and non-circadian photoperiods, and about half of them even in continuous darkness, while the females of the southern strains show high diapause proportions only in the 24 h photoperiod [45], see Figure 2(b). This shows that the northern strains possess a high stability against perturbations in the photoperiod length and in the presence of LD cycles than the southern ones.

### Latitudinal variation in fly cold tolerance, body colour and size

Distribution of *Drosophila* species in the Northern Hemisphere is largely determined by the flies’ ability to tolerate low temperatures [2,65,83], and some species, including *D. melanogaster* [84], have been shown to possess latitudinal variation in fly cold tolerance also within the species. Insects’ body colour typically becomes darker towards higher latitudes due to an increased ability of dark individuals to absorb solar radiation and warm up fast in cold environments with low solar radiation [85–87]. However, latitudinal clines in body colour may be complicated by the fact that this trait can also be affected by protection against UV-radiation [88], desiccation [89] and/or pathogens [90]. Latitudinal variation in insect body size is not always clear, either, as about half of the species show converse Bergman’s rule (get smaller towards North) instead of Bergman’s rule (get larger towards North), or show U-shaped clines, largely due to the short growing season on high latitudes limiting the time available for development, growth and foraging [91]. Overall, it is challenging to distinguish whether latitudinal clines in fly cold tolerance, body colour and size have evolved in response to changes in photoperiod (day length), temperature, or their combination [92], and whether variation in these traits is restricted or enhanced by trait correlations.

Poikela et al. [4] have studied latitudinal variation in the cold tolerance, body colour and size of *D. montana* and *D. flavomontana* flies in North America, paying special attention to the linkage of these traits with environmental variation. In this study, *D. montana* appeared to be more cold-tolerant than *D. flavomontana*, as would be expected on the basis of species distributions. Chill coma recovery time (CCRT) test showed the cold tolerance of both species to increase towards northern latitudes and in *D. montana* the chill coma temperature (CT_min_) showed association with latitudinally varying temperatures. Interestingly, *D. flavomontana* flies from the low-altitude western coast populations had a better basal cold tolerance than the ones from the high-altitude Rocky Mountains populations, which raises a question on whether the tolerance of the latter flies against cold conditions could be increased by better cold acclimation capacity. The body colour of *D. montana* flies was dark throughout the species distribution, while *D. flavomontana* flies were darkest in coastal populations and in the northern parts of the Rocky Mountains, and lightest in the southern Rocky Mountains in North America. Variation in *D. flavomontana* flies’ body colour showed no direct association with cold tolerance, and it would be interesting to see whether this trait is linked with desiccation tolerance as in some species of the *virilis* phylad [93] and/or with UV protection. Body size of both species decreased towards cold environments at population-level, even though large size correlated with fast recovery from chill coma (CCRT) within *D. montana* populations. The latter finding is consistent with our earlier observation that the overwinter survival of *D. montana* males increases along with an increase in body size in nature [51].

Wiberg et al. [94] found the genomic divergence of *D. montana* samples from two continents to be correlated with climatic variation and fly cold tolerance. In this study, the regions near SNPs associated with climatic variables were enriched for genes previously identified as candidates related to cold tolerance and diapause, especially on the X and fourth chromosomes [63,64,73,95]. Moreover, the study showed that some of the same biochemical processes that are targeted by selection on larger evolutionary scales (i.e. across species), are also involved in local adaptation within a species, which provides a rare bridge between adaptation and speciation.

### Number of generations per year (voltinism)

Insect life-cycles are adapted to seasonally varying climate by expressing alternative voltinism phenotypes, the number of generations per year generally declining towards higher latitudes [96,97]. Given that insects generally develop faster at higher temperatures, voltinism tends to covary not only with season length but also with mean temperature [97]. The optimal reproductive strategy for the univoltine northern insect populations should be an extended maturation period, during which an optimal dormancy fraction is evaluated independently every day [98]. Moreover, univoltine species and populations experience continual selection to adapt to changing seasonality, whereas the multivoltine ones experience reduced or no selection during the late season generations [99]. Seasonality may thus be a key factor promoting the evolution of seasonally polyphenic life histories [100].

Fine-scale variation in voltinism may be driven by local adaptation of photoperiodic plasticity, especially in species with strong photoperiodic regulation of CDL and development rate [101]. Due to the shortness of the warm season and the high risks associated with reproduction in late summer, high-latitude and high-altitude populations of *D. montana* produce only one full generation per year, while the southern ones are largely bivoltine [5]. Females of this species enter diapause already 2 to 3 months before the first frost, which gives their progeny enough time to develop and get prepared for the cold period. *D. montana* females’ egg-to-adult development time and their reproductive state at adulthood are determined by photoperiodic cues through different time measurement systems [59], and seasonal changes in the development time are difficult to predict as the fly development is fastest both under short-day conditions (late summer) and high temperatures (early summer) [60]. However, it is clear that uni- and bivoltine populations of this species experience quite different seasonal selection pressures in the wild.

## The evolution of reproductive isolation (RI) between diverging lineages

Speciation is often regarded as a gradual and slow process occurring over tens of thousands to millions of generations via natural and sexual selection and/or genetic drift, but it can also occur in a stepwise fashion reviewed in Kulmuni et al. [102]. Reproductive barriers between diverging lineages can be induced by any trait or mechanism that prevents or reduces their hybridization, and they can broadly be categorized into non-ecological and ecological premating barriers, postmating prezygotic (PMPZ) barriers and intrinsic and extrinsic postzygotic barriers reviewed in Coyne and Orr [6]. Premating reproductive barriers reduce matings between different taxa, while PMPZ arises from incompatibilities related to interactions between the sexes that act after copulation but prevent fertilization. Postzygotic barriers reduce the fitness of hybrid offspring, either due to genetic incompatibilities (intrinsic postzygotic isolation involving hybrid sterility or inviability) or due to the challenges that the hybrids meet when adapting to parental niches or finding a mating partner (extrinsic postzygotic isolation).

In a speciation by divergent selection – model, premating, PMPZ and extrinsic postzygotic barriers are predicted to evolve before the intrinsic postzygotic barriers, while a speciation by intrinsic barriers – model expects RI to be initially caused by intrinsic postzygotic barriers, after which selection favours the formation of strong prezygotic barriers to prevent the production of unfit hybrids reinforcement [103]. Yukilevich [104] performed a comparative study of male courtship songs of 119 *Drosophila* species across 10 distinct species groups and related song divergence to genetic distances, geographic relationships, and sexual isolation between species. This study showed that the species groups typically retain phylogenetic signal, while the species belonging to same group diverge five times more quickly in sympatry relative to allopatry, producing a pattern of reproductive character displacement. Moreover, enhanced prezygotic barriers against heterospecific individuals may strengthen prezygotic barriers against conspecific individuals living in a different region cascade reinforcement [105]. Coughlan and Matute [106] note that basically any trait that prevents costly parental investment in unfit hybrid offspring can become reinforced in sympatric populations, including ecological divergence and PMPZ barriers.

### North European *virilis* group species show strong reproductive isolation that is largely based on male songs

In the *virilis* group, the species of the *virilis* phylad have simple courtship songs consisting of dense pulse trains, while the rest of the species have species-specific songs [107]. Sexual isolation between the North European species of the group, *D. montana, D. littoralis, D. ezoana* and *D. lummei*, is largely based on species differences in male song. Even though interspecific courtships are common in the wild, they usually break down when the females begin to vibrate their wings as a sign of refusal in response to non-conspecific courtship song, and the same is true in laboratory experiments [108]. It is also extremely difficult to cross these species to produce interspecific hybrids in laboratory [14].

The role of male song in mate choice has been studied most intensively in *D. montana*, where the females do not accept the courting male without hearing his song [109]. In this species, the song plays an important role both in sexual selection [110] and species recognition [108,111]. Song simulation experiments performed in *D. montana* show that the carrier frequency and pulse length of the song play an important role in sexual selection [110], while interpulse interval is important both in sexual selection [112] and species recognition [111]. Ritchie et al. [113] also showed that selection for short sound pulses and high carrier frequency is directional, and that the song frequency and the female preference for it do not show temperature coupling (are not affected by temperature in a qualitatively similar fashion) in Finnish *D. montana. D. montana* males from a Colorado population produce a courtship song with a significantly higher carrier frequency than males from either a Canadian or a Finnish population, but the females of this population do not show preference for high-frequency song, which suggests that in this population the correlation between male fitness and his song frequency has changed and/or another trait has become a more reliable indicator of male fitness [19,114]. The difference in the song carrier frequency between *D. montana* males from Colorado and Finland involves a QTL with relatively large effect located near *fruitless* gene in a genomic region on the 2^nd^ chromosome partly covered by a polymorphic inversion [115,116].

### Reproductive barriers between *D. montana* and *D. flavomontana* in North America

**Figure 5.**
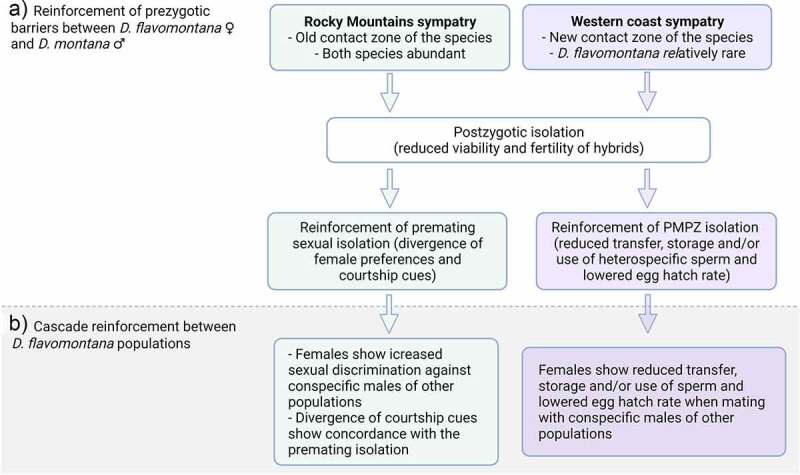
(a) Reinforcement of prezygotic (premating and PMPZ) barriers between *D. flavomontana* ♀ and *D. montana* ♂ in sympatric populations. (b) Cascade reinforcement between *D. flavomontana* populations that are or are not sympatric with *D. montana*. The figure was created with BioRender.com.

Reproductive isolation between *D. montana* females and *D. flavomontana* males is nearly complete, as *D. flavomontana* males fail to attract *D. montana* females and mate with them [117,118]. If *D. flavomontana* males occasionally succeed to mate with *D. montana* females, interspecific hybrids are not produced either due to strong PMPZ and/or intrinsic postzygotic isolation [118]. On the other hand, reproductive isolation between *D. flavomontana* females and *D. montana* males shows postzygotic barriers and the signatures of reinforcement of prezygotic barriers via increased mating discrimination, sexual signal divergence and PMPZ isolation in sympatric populations versus allopatric ones [118], Figure 5(a). The premating barriers in this direction are strongest in the Rocky Mountains, where the species have a long history of coexistence. In these populations, strong premating isolation is accompanied by high species- and sex-specific divergence in CHCs, which play an important role in *D. flavomontana* mate choice [118]. PMPZ barriers, on the other hand, are strongest in sympatric populations in the western coast, where *D. flavomontana* has arrived relatively recently and is still rare [118]. Strong PMPZ isolation was observed as decreased sperm storage and egg hatch rate, which could be due to incompatibilities between heterospecific gametes and/or between the female reproductive tract and male seminal fluids. Although the hybrids between *D. flavomontana* females and *D. montana* males have low fertility and viability (intrinsic postzygotic barriers), they can produce progeny by mating with the flies of the parental species [118] (Poikela et al., in prep.).

### Reproductive barriers between conspecific *D. montana* and *D. flavomontana* populations

*D. montana* populations from different geographic regions (Colorado and Vancouver populations from North America and Oulanka population from Finland) show partial sexual and PMPZ isolation, but no postzygotic isolation [119,120]. These populations differ in male courtship songs [19,114] and CHCs [121]), both of which may play a role in sexual isolation [120]. PMPZ isolation between Vancouver males and Colorado females is caused by the inability Vancouver males’ sperm to successfully fertilize Colorado females’ eggs [120], which could result from differences in male sperm length and female sperm storage organ morphology, interactions between sperm and egg cell surfaces and/or protein-level incompatibilities [18]. Interestingly, Garlovsky et al. [122] identified more than 150 differentially abundant male ejaculate proteins between *D. montana* populations.

*D. flavomontana* populations originating from different parts of North America show partial premating and PMPZ isolation [118]. Here, increased discrimination of *D. flavomontana* females against *D. montana* males in sympatric populations may have facilitated reproductive barriers also between conspecific *D. flavomontana* populations living in and outside the areas of sympatry cascading reinforcement [105]. Cascading reinforcement has happened in the same barriers as the reinforcement of barriers between *D. flavomontana* females and *D. montana* males: females from the Rocky Mountains population show increased sexual (premating) isolation and the ones from the western coast increased PMPZ barriers towards the males of other populations (Figure 5(b)). Divergence in the CHCs of *D. flavomontana* populations living in different climatic conditions could also have been enhanced by natural selection, but this cannot explain higher sex differences in CHCs in sympatric compared to allopatric populations [118].

## Chromosomal inversions play a prominent role in adaptation and speciation processes

Chromosomal inversions can be highly beneficial both in adaptation and speciation, when they protect locally adapted allele complexes from the homogenizing effects of recombination and gene flow [12]. Such inversions are particularly good candidates for the onset of the speciation process, since multiple substitutions driven by selection are ultimately expected to lead to enough divergence to cause genetic incompatibilities [11,123]. Because inversions suppress recombination, an incompatibility allele residing within an inversion can effectively impede gene flow between taxa over a larger region than an allele locating in a collinear region [124]. If an inversion causes intrinsic postzygotic isolation, then selection may also favour alleles that increase the strength of prezygotic isolation reinforcement [125]. This idea is already supported by experimental evidence reviewed in Faria and Navarro [126], but recent advances on genomic sequencing and demographic modelling are expected to give more information on the role of inversion in speciation.

Chromosomal inversions of the *virilis* group species have been studied intensively in 1950s, e.g. by [127–130]. According to Throckmorton [14], the species of the *montana* phylad (presently *montana* and *littoralis* phylads) have evolved more in terms of fixed inversions, and are more variable in terms of the number of segregating inversions than the species of the *virilis* phylad. *D. montana* exhibits a high number of fixed and polymorphic inversions, and American populations of this species have been classified as Standard (S), Giant (G) and Alaskan-Canadian (AC) *montana* on the basis of their geographical origin, size and chromosome structure, see Throckmorton [14]. Morales-Hojas et al. [131] also characterized chromosomal inversion polymorphism in Finnish *D. montana* populations and found a total of 14 polymorphic inversions, nine of which had not been described before. Interestingly, in a wide variety of species, including the *virilis* group species, transposable elements (TEs) have been found play a significant role in promoting the formation of inversions and other large and small chromosomal rearrangements (e.g. [132]). Recent studies have also shown that ectopic recombination (nonallelic homologous recombination between multiple copies of DNA sequences present in the same chromosome in opposite directions) may be the prevalent mechanism of generating inversions at least in the *virilis* phylad species [133,134]. Here, DAIBAM (a miniature inverted–repeat TE) seems to be responsible for a large number of inversions observed within and between species of this phylad [133,134].

### Species-specific inversions have enhanced divergence and speciation of *D. montana* and *D. flavomontana*

A recent study by Poikela et al. (in prep.) shows that most of the large (>1 Mb) wide-spread species-specific inversions of *D. montana* and *D. flavomontana* on the X, 2 L, 4 and 5 chromosomes have arisen around or before the species split, and that they had existed as polymorphisms already in the ancestral lineage of the species. Increased genetic divergence in neutral (non-coding) sequences in inverted compared to non-inverted (colinear) genomic regions and the lack of extensive introgression, detected in this study, suggest that the inversions have played a major role in reducing recombination and gene flow between the local populations of the ancestral form. Over time, inverted regions can have accumulated genetic incompatibilities and other reproductive barriers to prevent maladaptive hybridization (Poikela et al., in prep.). Inversions on the X and 4^th^ chromosomes are particularly good candidates for enhancing ecological divergence and early reproductive isolation between *D. montana* and *D. flavomontana*, since they show elevated genetic divergence on coding sequences (Poikela et al., in prep.). Moreover, these chromosomes are known to involve genes that are important in diapause induction [135] and cold tolerance [94], and X chromosomal genes have also played an important role in the evolution of species-specific courtship songs [107,136].

To find out whether the chromosomal inversions harbour genetic incompatibilities, Poikela et al. (in prep.) performed repeated backcrosses for female hybrids between *D. flavomontana* females and *D. montana* males in laboratory. In this study, fertile F_1_ females were backcrossed towards both parental males for two generations, and the resulting second-generation backcross (BC2) females were pool-sequenced to quantify reductions in interspecific gene flow (introgression) due to genetic incompatibilities. The backcrossing towards *D. flavomontana* showed that introgression was strongly reduced throughout the X chromosome, likely due to a strong incompatibility allele residing within several overlapping inversions. The most highly diverged, non-introgressed genes were associated with embryo development, meiosis and/or gametogenesis (oogenesis and spermatogenesis), and are thus good candidates for causing genetic incompatibilities in hybrids. Such strong postzygotic isolation might have led to the reinforcement of prezygotic barriers, like the one observed between *D. flavomontana* and *D. montana* by Poikela et al. [118].

## Conclusions

*Drosophila* species offer a treasure-chest for the researchers of several fields. *D. melanogaster* has long been a superior study organism for genetic research, and a wide range of genetic tools developed for this species can nowadays be used also for its less well-known relatives. On the other hand, *Drosophila* species that are adapted to live in a wide-range of environmental conditions on different latitudes, including some species of the *virilis* and *obscura* groups, offer great possibilities to study the role of natural and sexual selection in species and population divergence. As shown in the present review, knowing the habitat requirements of the flies and collecting fresh study material from different parts of the species distribution gives a firm basis for this kind of studies. It is also important to maintain fly strains in laboratory in lighting and temperature conditions, where the flies reproduce well and where their time-measuring systems preserve the characters typical to fly home population [82]. This kind of material offers unique opportunities to trace linkages between fly reproduction and stress-tolerances with photoperiodic and/or climatic conditions in the wild, to examine possible interaction between natural and sexual selection in building up reproductive barriers between the diverging taxa and to trace genetic changes underlying adaptation and speciation.

Sequencing the genomes of wild-collected flies has boosted the development of computational methods, which enable researchers to trace the signs of local adaptation and lineage divergence millions of years backwards and to identify transposable elements (TEs), chromosomal inversions and even parasites present in the study organisms. Our ongoing studies on polymorphic inversions and TEs of *D. montana* throughout its distribution range are likely to provide new insights into the genomic factors underlying adaptation to variable climates and photoperiods both in time and space. Moreover, the use of modern gene editing techniques, especially when combined with feasible ecological and experimental studies, could allow more precise identification of the genes and gene complexes underlying hybrid incompatibilities. For example, CRISPR/Cas9 gene editing technique could be used to revert the X chromosomal inversions in *D. montana* and *D. flavomontana*, after which the causal genes for hybrid incompatibilities could be identified through repeated hybridization and resequencing, as suggested by Hopkins et al. [137].

Global climate change increases average temperatures and induces heat waves, which shuffles the association between photoperiod and temperature especially at high latitudes and induces changes in species distribution and coexistence. Moreover, the complexity of insect life-history, fertility and stress tolerance traits at genetic, metabolic and neuronal level, as well as their correlations, imposes big challenges for the evolvability of these traits in changing environmental conditions. Understanding the changes occurring in wild populations requires versatile studies on insect species with different kinds of life-histories and reproduction strategies, as well as sharing of research ideas and material between the specialists on different fields.
